# A Treatise on Operative Ophthalmic Surgery

**Published:** 1853-10

**Authors:** 


*A Treatise on Operative Ophthalmic Surgery.* By H. HAYNES WALTON, Fellow of the Royal College of Surgeons in England, etc., etc. First American from the first London edition. Illustrated by one hundred and sixty-nine engravings on wood. Edited by S. LITTELL, M. D., author of a Manual of the Diseases of the Eye, etc., etc. Philadelphia: Lindsay & Blakiston. 1853. 8vo. pp. 599.

Of this new candidate for favor the American editor thus speaks:—"It is evidently the production of a thoughtful and independent observer, thoroughly acquainted with his subject, and capable of imparting to others, clearly and plainly, the knowledge which he possesses." "It embodies the results of extensive literary research and close personal observation, under circumstances of rare opportunity."

The work has been highly commended by English reviewers.

For sale by DERBY, ORTON & MULLIGAN.

